# Cement bridging phenomenon in percutaneous vertebroplasty for adjacent vertebral compression fracture

**DOI:** 10.1038/s41598-021-89412-z

**Published:** 2021-05-13

**Authors:** Yun-Da Li, Tsung-Ting Tsai, Chi-Chien Niu, Po-Liang Lai

**Affiliations:** 1 Department of Orthopedic Surgery, Spine Section, Chang Gung Memorial Hospital, No. 5, Fusing St., Guishan Dist., Taoyuan, 33305 Taiwan; 2 Bone and Joint Research Center, Chang Gung Memorial Hospital, Taoyuan, Taiwan; 3 College of Medicine, Chang Gung University, Taoyuan, Taiwan; 4 Department of Orthopedic Surgery, New Taipei Municipal TuCheng Hospital, Chang Gung Memorial Hospital, Taoyuan, Taiwan

**Keywords:** Musculoskeletal system, Geriatrics, Fracture repair, Trauma, Bone imaging, Magnetic resonance imaging, Spine structure

## Abstract

In some cases of vertebroplasty for adjacent fractures, we observed a cement bridging phenomenon, in which the injected cement flowed from the newly fractured vertebra to the previously cement-augmented vertebra through the space between the abutting anterior longitudinal ligament and the vertebral column. The purpose of this retrospective study was to investigate this phenomenon. From January 2012 to December 2014, patients who sustained new-onset adjacent vertebral compression fracture and who were again treated with vertebroplasty were enrolled. We divided the patients into two groups, the bridging group and the nonbridging group, to analyze the difference between them. Results showed that the cement bridging phenomenon occurred in 18 (22.8%) of the 79 patients. Significant differences between the bridging and nonbridging groups were identified in the following 3 imaging features: severe loss of the anterior vertebral body height at the new-onset adjacent vertebra on plain film (odds ratio [OR] = 4.46, p = 0.014), fluid accumulation (OR = 36.27, p < 0.001) and hypointense signaling (OR = 15.67, p < 0.001) around the space anterior to the abutting vertebral bodies and the corresponding intervertebral disc on MRI. After a 2-year follow-up, both the mean value of the focal kyphotic angle and anterior body height ratio were significantly better in the cement bridging group than in the nonbridging group. The cement bridging phenomenon, which has never been reported in the literature, is not rare in clinical practice. This phenomenon was associated with better maintenance of focal kyphotic angle and anterior body height ratio during the 2-year follow-up.

## Introduction

Percutaneous vertebroplasty was first introduced in 1984 in France by Galibert for the treatment of a painful hemangioma at the C2 vertebral body^1^. Since then, this procedure has been used extensively worldwide for the treatment of osteoporotic vertebral compression fracture (VCF). The advantages of percutaneous vertebroplasty are that it is minimally invasive and efficient not only for providing pain relief and spinal stabilization but also for reducing the side effects of medications and the risks of long-term limited mobility^2–4^.

However, some complications remain, such as cement leakage, pulmonary embolism and new-onset vertebral compression fracture^5–7^. The incidence of subsequent new-onset VCF after percutaneous vertebroplasty has been reported as 6.5–52% in the literature, usually occurring in adjacent vertebral bodies^7–11^. Secondary cement augmentation is an effective procedure for treating adjacent VCF^12^.

**Figure 1:**
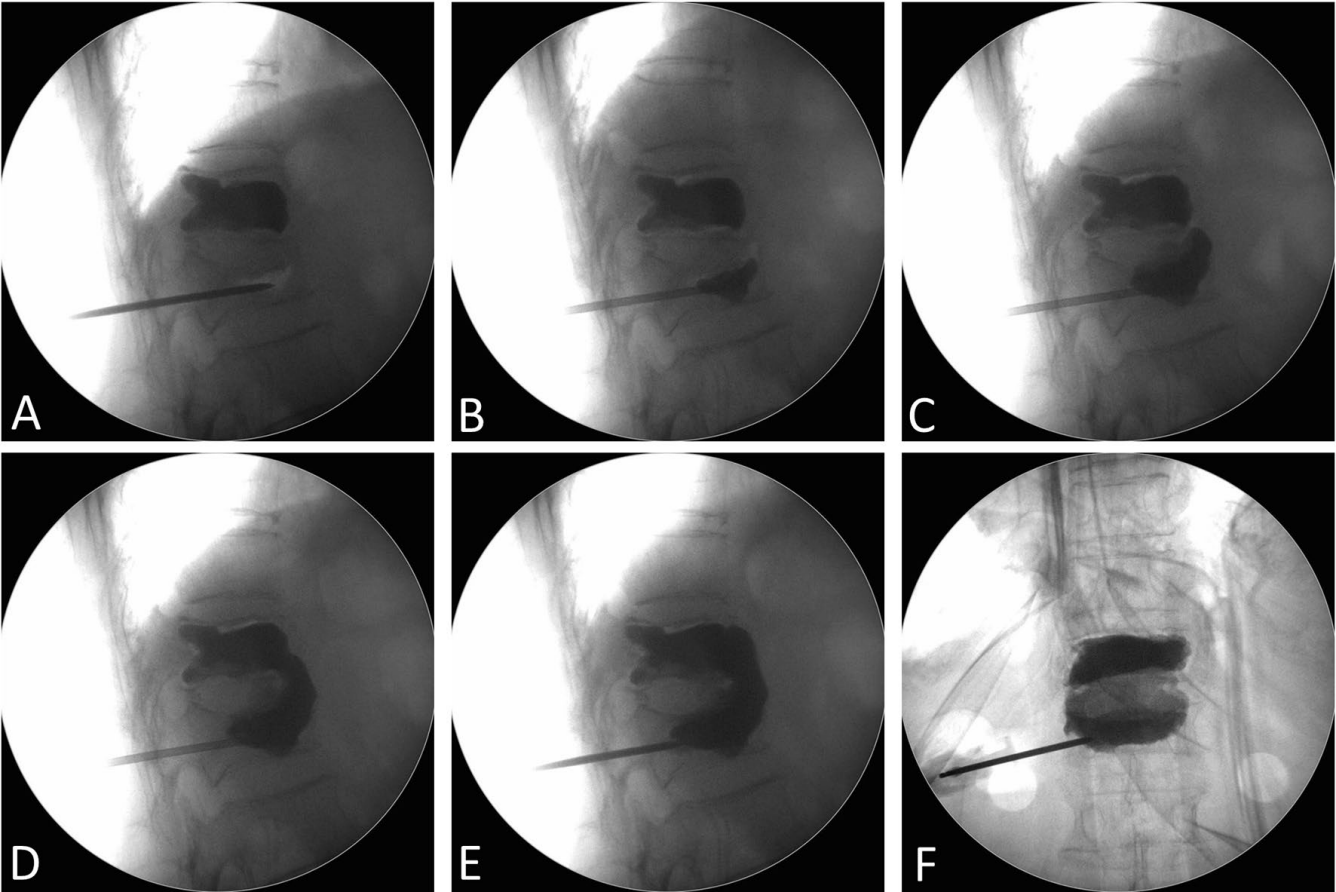
(**A**–**F**) Serial intraoperative fluoroscopy images that illustrated the cement bridging phenomenon, in which the injected cement filling the newly fractured vertebra flowed through the anterior margin of the spinal column and then reached the previously cement-augmented vertebra.

During the procedure of percutaneous vertebroplasty for new-onset adjacent vertebral compression fracture, we observed a cement bridging phenomenon (CBP), in which the injected cement flowed from the newly fractured vertebra to the previously cement-augmented vertebra through the space between the abutting anterior longitudinal ligament and the vertebral column (Fig. 1). This phenomenon has never been described in the literature. Therefore, the purpose of our study was to elucidate this phenomenon, and further analyze the imaging predictors and clinical significance of the cement bridging phenomenon.

## Materials and methods

### Data collection

Between January 2012 and December 2014, all the patients in our institute were included in this study if they: (1) sustained new-onset adjacent vertebral compression fractures; and (2) were treated again with vertebroplasty. The local institutional review board approved this retrospective study (Institutional Review Board of Chang Gung Medical Foundation Reference Number: 201900794B0) and waived the requirement for written informed patient consent. All methods were conducted in accordance with the relevant guidelines and regulations.

The cement bridging phenomenon was defined as the presence of injected cement filling the newly fractured vertebra, flowing through the anterior margin of the spinal column, and then reaching the previously cement-augmented vertebra. The CBP was observed by orthogonal intraoperative fluoroscopy and confirmed by postoperative plain films. Each image was independent interpretated by two authors (YD Li and PL Lai) to reach agreement on the presence or absence of the CBP in one patient.

**Figure 2:**
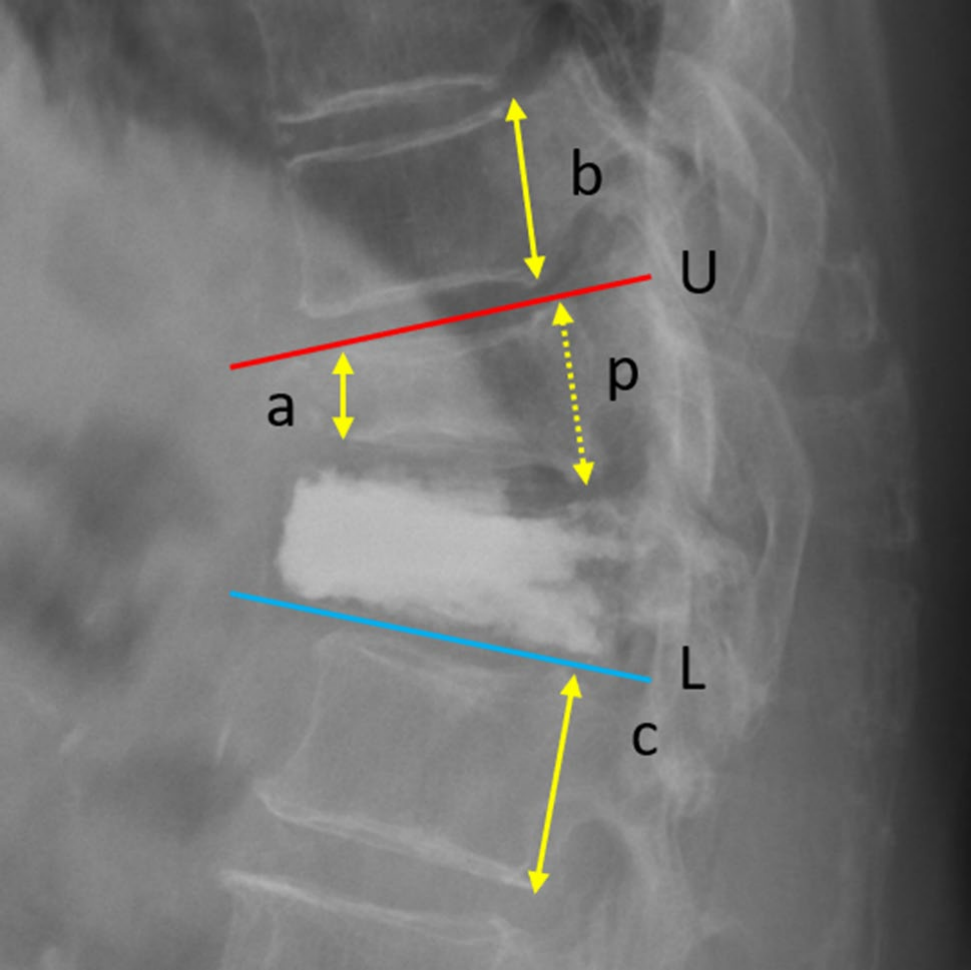
Measurement of the anterior body height and focal kyphotic angle. a, anterior body height of the VCF; b, posterior vertebral height of the adjacent cranial vertebra; c, posterior vertebral height of the adjacent caudal vertebra; p, estimate of the posterior body height of the VCF (average of b and c). The anterior body height ratio is a/p; U, the line parallel to the upper endplate of the cranial vertebra (red line); L, the line parallel to the lower endplate of the caudal vertebra (blue line). The focal kyphotic angle is the intersecting angle of U and L.

Preoperative patient characteristics, including age, sex, body mass index (BMI), T-score of bone mineral density (BMD), findings from preoperative radiographic studies and MRI data, were recorded. The preoperative MRI analysis focused on both fluid accumulation or hypointense signaling around the space anterior to the abutting vertebral bodies and the corresponding intervertebral disc. The details of vertebroplasty were investigated, including the volume of injected cement and patterns of cement distribution. Postoperative data were reviewed, including the incidence rates of further adjacent fractures, serial changes in the anterior body height ratio of the newly fractured vertebra and the focal kyphotic angle (Fig. 2), and clinical outcomes (based on the frequency of analgesics prescription in the outpatient clinics) within 2 years. We divided the patients into two groups, the bridging group (with the cement bridging phenomenon) and the nonbridging group, to analyze the difference between them.

### Operative procedures

At the beginning of the procedure, the patient was placed in the prone position on the operating table. The surgical field and surgical drapes were carefully disinfected. After proper administration of local anesthesia, a vertebroplasty needle was inserted into the vertebral body through the pedicle under fluoroscopic guidance. After the needle was gently hammered into the anterior third of the vertebral body, preparation for the polymethylmethacrylate (PMMA) bone cement injection was initiated. The injection timing was chosen between the liquid phase and the paste phase of the bone cement. The injection was performed until symmetrical filling of the central and anterior parts of the vertebral body was obtained. Special attention was given to the flow of the bone cement while cement was distributed across the anterior margin of the spinal column.

While the cement bridging phenomenon developed, the cement was continuously and cautiously injected. Injection typically ceased when cement reached the posterior quarter of the vertebral bodies (either the newly fractured vertebra or previously cement-augmented vertebra), leaked into veins, or leaked laterally out of osseous structures.

### Postoperative care

It was suggested that all patients wear an orthosis when ambulating for 3 months. In general, antiosteoporosis medications and calcium and vitamin D supplementation were prescribed for the patients after surgery for osteoporotic VCFs. However, because of the potential adverse interaction with coexisting morbidities, antiosteoporosis medication was used on a case-by-case basis. These patients were usually discharged on the day of surgery or the next day and followed-up at 1 and 3 months postoperatively and then annually at outpatient clinics.

### Statistical analysis

Associations between the cement bridging phenomenon and potential predictors were analyzed by the chi-square test or Fisher’s exact test for categorical variables and Student’s t test for continuous variables. Logistic regression analysis was used for the calculation of odds ratios and 95% confidence intervals. A *p* value < 0.05 was considered significant. Statistical analysis was performed using SPSS 25.0 (IBM, Armonk, New York). Power analysis was performed using the following parameters: test family—t tests, difference between two independent means (two groups), two-tailed, α = 0.05. (G*Power 3.1.9.4, University of Kiel, Germany).

## Results

### Patient characteristics and imaging features

A total of 79 patients (94 vertebrae) sustained new-onset adjacent VCF and then underwent further vertebroplasty. In 18 of 79 (22.8%) patients, or 20 of 94 (21.3%) adjacent vertebrae, the cement bridging phenomenon was observed. The mean age of patients who developed adjacent VCFs was 78.0 ± 6.3 years (range 66–95 years), and female patients made up the majority (87%). The most commonly involved vertebral level was T12 (36.2%), followed by L1 (21.3%) and L2 (16.0%).

**Figure 3:**
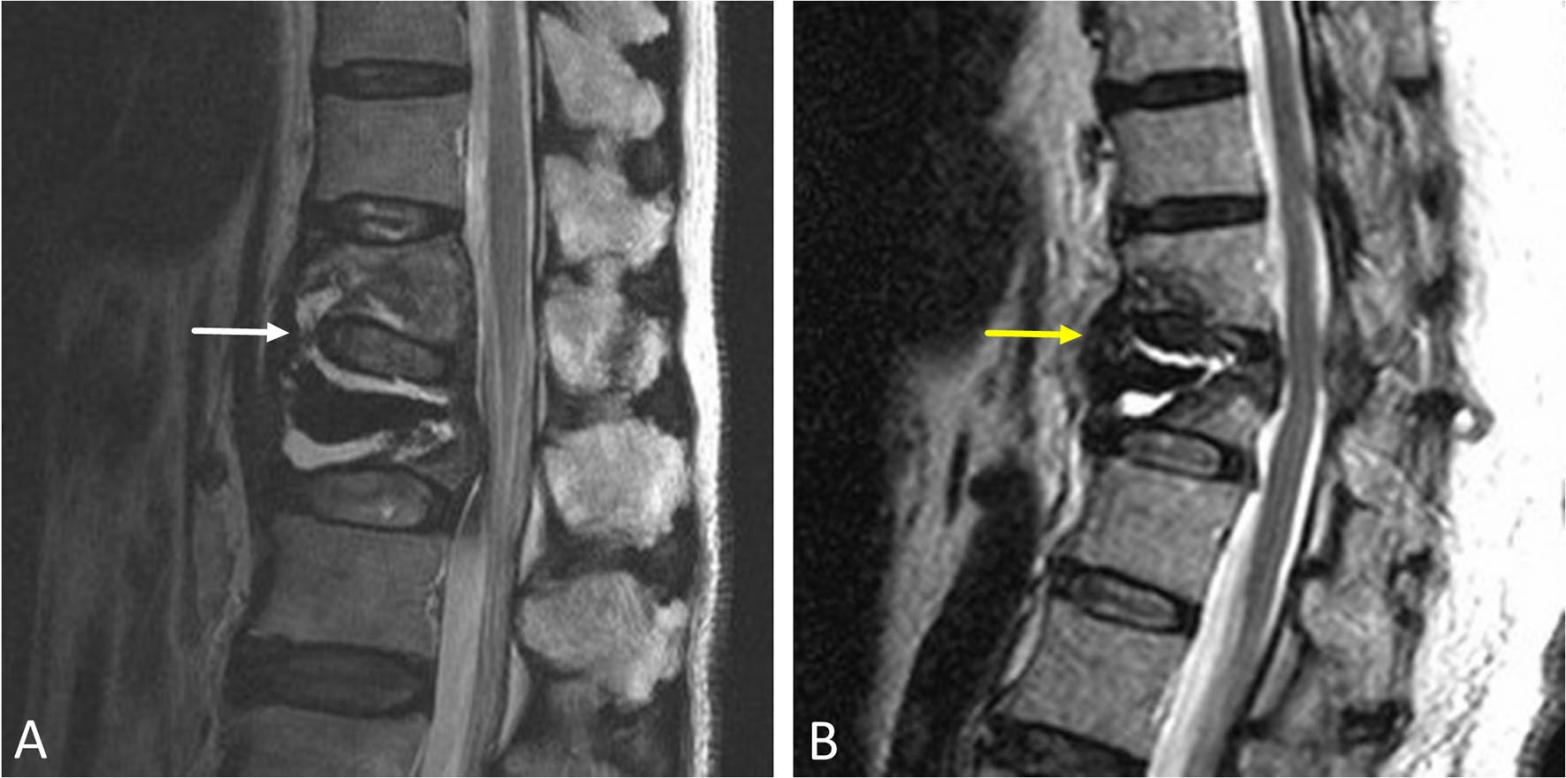
Preoperative MRI predictors of the cement bridging phenomenon include (**A**) fluid accumulation (white arrow) and (**B**) hypointense signaling (yellow arrow), indicating a redundant anterior longitudinal ligament, around the space anterior to the abutting vertebral bodies and the corresponding intervertebral disc.

The demographic data and the imaging features in the bridging and nonbridging groups are listed in Table 1. Significant differences were found with regard to three preoperative imaging features (*p* < 0.05), which included severe loss (more than 50%) of the anterior vertebral body height at the new-onset adjacent vertebra on a lateral-view plain film (odds ratio [OR] = 4.46, *p* = 0.014), fluid accumulation (OR = 36.27, *p* < 0.001) or hypointense signaling (OR = 15.67, *p* < 0.001) around the space anterior to the abutting vertebral bodies and the corresponding intervertebral disc on MRI (Fig. 3). Furthermore, 14 vertebrae (70%) that were previously cement-augmented vertebrae demonstrated increased cement volume due to the cement bridging phenomenon. No statistical significance in patient age, sex ratio, BMI, T-score of BMD or injected cement volume was found between the bridging and nonbridging groups (*p* > 0.05).

**Table 1:** Patient characteristics and imaging features of the bridging and nonbridging groups.

	Bridging group (n = 20)	Nonbridging group (n = 74)	OR (95% CI)	*p* value
Age (years)	79.1 ± 7.3	77.8 ± 6.0	0.97 (0.89–1.05)	0.414
Sex (female)	20 (100%)	63 (85%)	N/A	0.965
BMI	21.1	21.6	0.93 (0.77–1.13)	0.478
T-score of BMD	− 3.12	− 3.02	0.91 (0.47–1.78)	0.789
Cement volume (ml)	7.3	6.2	0.83 (0.68–1.02)	0.072
Severe anterior body height loss on X-ray*	16	35	4.46 (1.36–14.61)	0.014
Fluid accumulation on MRI**	17	10	36.27 (8.97–146.58)	< 0.001
Hypointense signal on MRI***	18	27	15.67 (3.37–72.76)	< 0.001
Cement volume increase****	14	0	N/A	< 0.001

*Severe loss (more than 50%) of anterior vertebral body height at the new-onset adjacent vertebra on X-ray.

**Fluid accumulations around the space anterior to the abutting vertebral bodies and the corresponding intervertebral disc on MRI.

***Hypointense signal around the space anterior to the abutting vertebral bodies and the corresponding intervertebral disc on MRI.

****Cement volume increase in the previously augmented vertebra.

BMI body mass index, BMD bone mineral density, OR odds ratio, CI confidence interval, N/A not applicable.

There was no major complication that was directly attributable to vertebral augmentation in the bridging and nonbridging groups, such as epidural hematoma, pulmonary emboli, or cement leakage into the spinal canal.

### Follow-up and clinical correlation

A total of 18 patients (20 vertebrae) in the bridging group and 56 patients (68 vertebrae) in the nonbridging group who had followed up for at least 2 years were enrolled for further analysis of clinical correlations.

No statistical significance in the incidence rate of adjacent fractures was found between the bridging and nonbridging groups during 2-year follow-up. The incidence rates of adjacent fractures in the bridging group were 15.0%, 20.0%, and 20.0% at 3 months, 1 year, and 2 years postoperatively, respectively. The incidence rates of adjacent fractures in the nonbridging group were 14.7%, 17.7%, and 19.1% at 3 months, 1 year, and 2 years postoperatively, respectively.

Compared with the nonbridging group, the cement bridging group demonstrated a significantly better mean value of the focal kyphotic angle (*p* < 0.05) at 3 months, 1 year, and 2 years postoperatively. The mean anterior body height ratio of the newly fractured vertebra was also significantly better (*p* < 0.05) in the cement bridging group than in the nonbridging group 1 year and 2 years postoperatively. The detailed values are listed in Table 2.

**Table 2:** Two-year follow-up of the incidence rate of adjacent fractures and radiographic parameters in the bridging and nonbridging groups.

		Bridging group (n = 20)	Nonbridging group (n = 68)	*p* value
Adjacent fracture	Postop 3 months	3	10	0.974
	Postop 1 year	4	12	0.810
	Postop 2 years	4	13	0.930
Mean focal kyphotic angle (degrees)	Preop	21.2∘	21.9∘	0.774
	Postop 1 day	15.6∘	18.9∘	0.173
	Postop 3 months	17.1∘	26.4∘	0.012
	Postop 1 year	18.0∘	27.3∘	0.017
	Postop 2 years	19.9∘	29.4∘	0.007
Mean anterior body height ratio	Preop	0.688	0.683	0.850
	Postop 1 day	0.745	0.718	0.324
	Postop 3 months	0.730	0.678	0.119
	Postop 1 year	0.709	0.636	0.026
	Postop 2 years	0.692	0.610	0.005

Preop preoperative, postop postoperative.

Postoperatively, patients were scheduled to return for follow-up at 1 and 3 months after the surgery and then annually at outpatient clinics. However, some patients might visit the clinic more frequently than the scheduled follow-up if pain sustained. The number of patients who visited the outpatient clinics as scheduled follow-up and more frequently than the scheduled follow-up for the bridging group (n = 18) were 12 and 6; for the nonbridging group (n = 56) were 39 and 17, respectively. No statistical significance in the frequency of analgesics prescription in the outpatient clinics was found between the bridging and nonbridging groups during 2-year follow-up (*p* = 0.812).

Power analysis showed that under the setting of α = 0.05 and current sample size, the power of our study about the three preoperative imaging features and two postoperative radiographic parameters at 2-year follow-up were all more than 0.9.

## Discussion

The cement bridging phenomenon (CBP) is sometimes observed during the procedure of cement augmentation but has, to the best of the authors’ knowledge, not been published before. Due to the unknown mechanism and cause of formation, the cement bridging phenomenon usually presents unpredictably. When the phenomenon occurred, we were unclear about the clinical manifestation of this phenomenon. To solve this question, we tried to describe this phenomenon clearly, investigate the predictors of occurrence, and correlate the results with clinical application.

The severe loss (more than 50%) in the anterior vertebral body height at the new-onset adjacent vertebra on plain film, fluid accumulation and hypointense signaling around the space anterior to the abutting vertebral bodies and the corresponding intervertebral disc on MRI were the three predictors of the CBP identified in this study. These three characteristics were presumed to be related to structural disruption anterior to the vertebral bodies and the corresponding intervertebral disc, indicating disruption of the anterior cortex and detachment of the anterior longitudinal ligament. The severe loss of anterior vertebral body height at the new-onset adjacent vertebra and hypointense signaling changes around the space anterior to the abutting vertebral bodies and the corresponding intervertebral disc on MRI suggest redundancy of the anterior longitudinal ligament. Therefore, an anterior intervertebral space existed, which may be related to the phenomenon of bone cement flowing from this space. Fluid accumulation around the space anterior to the abutting vertebral bodies and corresponding intervertebral disc results in the possibility of bone cement flowing from this space as well.

The cement bridging phenomenon is essentially an event of cement leakage because cement leakage has been defined as the presence of any extravertebral cement. Yeom et al.^13^ proposed three patterns of cement leakage: (1) leakage via the basivertebral vein (B-type), (2) leakage via the segmental vein (S-type), and (3) leakage through a cortical defect (C-type). The cement bridging phenomenon develops leakage through a cortical defect, belonging to the C-type. Nieuwenhuijse et al.^5^ reported that the incidence of cortical leakage increased with advancing severity grade, which is consistent with the results of our research, that is, the severe loss of anterior vertebral body height was highly associated with the CBP.

Some studies^14–17^ have shown that cement leakage into a disk is associated with adjacent VCFs. This made us wonder whether the CBP would also have a similar effect. However, there was no statistical significance in the incidence rates of secondary adjacent fracture between the bridging and nonbridging groups during the 2-year follow-up in our study. In fact, this issue is still inconclusive because several studies^4,18,19^ have demonstrated leakage unrelated to new fractures either at adjacent or non-adjacent levels. A meta-analysis conducted by Yi Zhan et al.^20^ mentioned that cement extravasation can also lead to serious complications including neurologic deficit and fetal sequalae such as pulmonary embolism, but it is generally of no clinical symptoms. Hassan et al.^21^ reported that there was no significantly difference in patient satisfaction between those who had cement extravasation and those who did not, in both groups.

In spite of no major complication with regarding to the cement bridging phenomenon in our study, there was one case who developed lateral cement extravasation into paraspinal soft tissue during cement bridging phenomenon. This patient felt temporary paraspinal burning sensation during the cement curing process, and this symptom subsided completely after the procedure. In our opinion, leakage of bone cement to the lateral side is usually far more prone to produce symptoms than leakage to the front area. Faded color of the injected cement during the injection in the lateral view was usually a sign of lateral extravasation.

Fourteen vertebrae (70%) that were previously cement-augmented demonstrated increased cement volume in the bridging group, which means that injected cement filled the newly fractured vertebra and the previously cement-augmented vertebra simultaneously via the cement bridging phenomenon. This finding was presumed to be related to the concurrent refracture of the previously cement-augmented vertebra and represented the real existence of the cement bridging phenomenon. Repeated percutaneous vertebroplasty for refracture of cemented vertebrae is a feasible treatment^22^, but an alternative method is to fill these two neighboring vertebrae through one portal via the cement bridging phenomenon. To achieve this condition, we recommended to insert needles in these two neighboring vertebrae concurrently before cement injection. Then, we suggested to inject cement through the needle in the newly fractured vertebra first. Once CBP and cement filling in these refractured vertebrae developed, cement can be continuously injected with caution. If satisfactory filling pattern in the refractured vertebra was already achieved by this way, no further injection through the needle in the refractured vertebra was needed.

**Figure 4:**
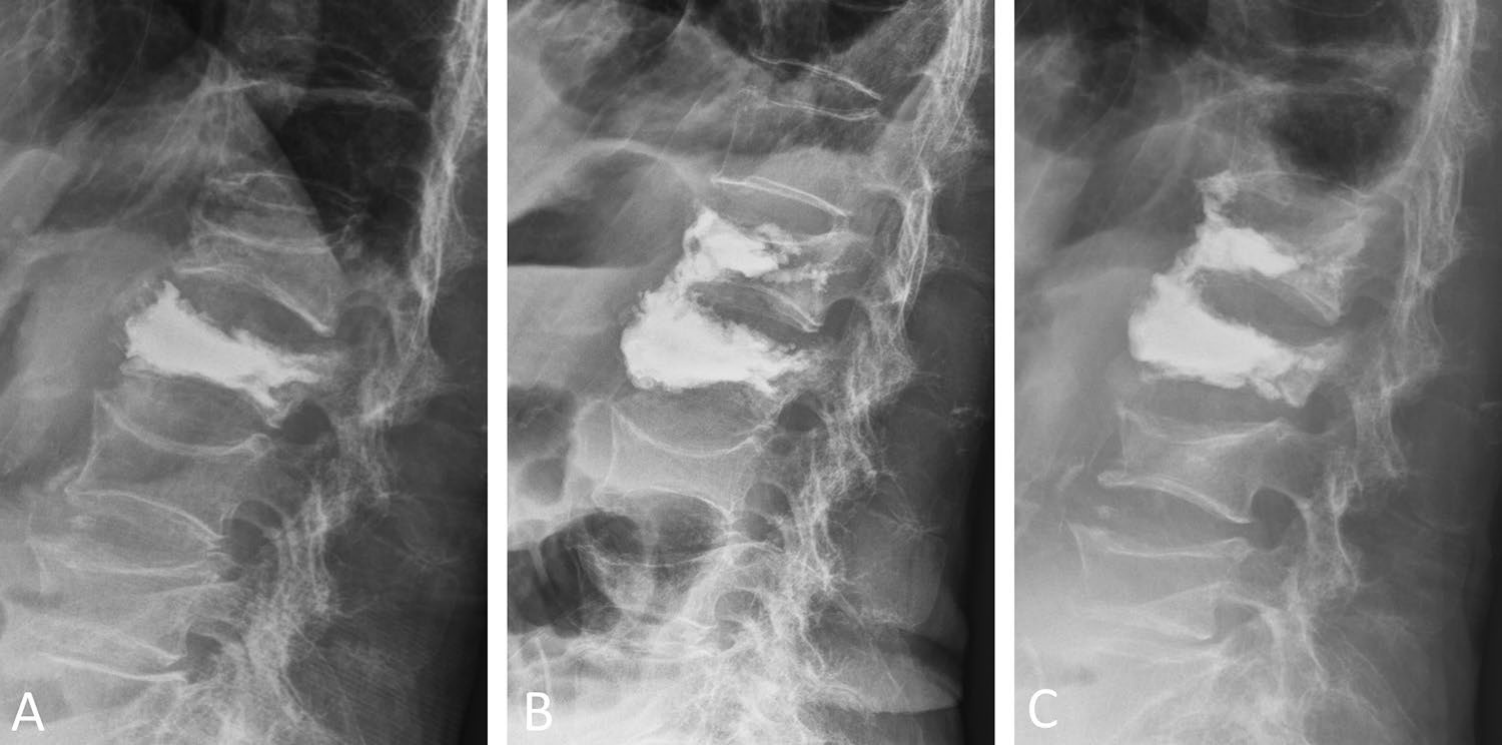
(**A**) A 95-year-old woman sustained a new-onset adjacent L1 compression fracture 3 weeks after L2 vertebroplasty, (**B**) the cement bridging phenomenon was observed during L1 vertebroplasty. Increased cement volume of the previously cement-augmented L2 vertebrae was noted compared with the preoperative image. (**C**) Two years postoperatively, the focal kyphotic angle and bony alignment were maintained.

The cement bridging phenomenon may offer anterior support of the vertebral column. Both the mean value of the focal kyphotic angle and mean anterior body height ratio were significantly better (*p* < 0.05) postoperatively in the cement bridging group than in the nonbridging group during the 2-year follow-up (Fig. 4). We think that it is difficult to promote and is unnecessary to pursue the cement bridging phenomenon. However, we should not worry this phenomenon when it occurs.

There are several limitations in this retrospective study, including the relatively small number of new-onset VCFs in the bridging group and the different end points of the operator during cement injection, which may result in different cement distribution patterns by different operators even under the same conditions. We did not analyze the influence on cement bridging between vertebroplasty and kyphoplasty, because most surgeons performed vertebroplasty in our institute. In addition, we did not objectively measure the bone cement viscosity, although the injection timing was consistently chosen between the liquid phase and paste phase. We think this method can minimize the possible impact of this factor.

## Conclusion

The cement bridging phenomenon (CBP), which has never been reported in the literature, is not rare in clinical practice. The severe loss of anterior vertebral body height at the new-onset adjacent vertebra, fluid accumulation and hypointense signaling around the space anterior to the abutting vertebral bodies and the corresponding intervertebral disc on MRI were identified as predictors of the CBP. The cement extravasation could lead to complications, but may offer positive effect with occurrence of CBP through cautious control of cement injection. Cement bridging phenomenon was associated with better maintenance of focal kyphotic angle and anterior body height ratio during the 2-year follow-up.

## Abbreviations

CBP: Cement bridging phenomenon
OR: Odds ratio
PMMA: Polymethylmethacrylate
VCF: Vertebral compression fracture
BMI: Body mass index
BMD: Bone mineral density
